# Use of 3D Printing in Preoperative Planning and Training for Aortic Endovascular Repair and Aortic Valve Disease

**DOI:** 10.21470/1678-9741-2018-0101

**Published:** 2018

**Authors:** Eduardo Nascimento Gomes, Ricardo Ribeiro Dias, Bruno Aragão Rocha, José Augusto Duncan Santiago, Fabrício José de Souza Dinato, Eduardo Keller Saadi, Walter J. Gomes, Fabio B. Jatene

**Affiliations:** 1 Cardiovascular Surgery Division, Instituto do Coração do Hospital das Clínicas da Faculdade de Medicina da Universidade de São Paulo (InCor-HCFMUSP), São Paulo, SP, Brazil.; 2 Instituto de Radiologia do Hospital das Clínicas da Faculdade de Medicina da Universidade de São Paulo (InRad-HCFMUSP), São Paulo, SP, Brazil.; 3 Department of Cardiovascular Surgery, Hospital de Clínicas de Porto Alegre (HCPA), Universidade Federal do Rio Grande do Sul (UFRGS), Porto Alegre, RS, Brazil.; 4 Cardiology and Cardiovascular Surgery Disciplines, Escola Paulista de Medicina da Universidade Federal de São Paulo (EPM-UNIFESP), São Paulo, SP, Brazil.

**Keywords:** Aorta/Surgery, Aorta, Thoracic, Endovascular Procedures, Aneurysm, Aneurysm, Dissecting, Imaging, Three-Dimensional, Models, Cardiovascular

## Abstract

**Introduction:**

Three-dimensional (3D) printing has become an affordable tool for assisting heart surgeons in the aorta endovascular field, both in surgical planning, education and training of residents and students. This technique permits the construction of physical prototypes from conventional medical images by converting the anatomical information into computer aided design (CAD) files.

**Objective:**

To present the 3D printing feature on developing prototypes leading to improved aortic endovascular surgical planning, as well as transcatheter aortic valve implantation, and mainly enabling training of the surgical procedure to be performed on patient's specific condition.

**Methods:**

Six 3D printed real scale prototypes were built representing different aortic diseases, taken from real patients, to simulate the correction of the disease with endoprosthesis deployment.

**Results:**

In the hybrid room, the 3D prototypes were examined under fluoroscopy, making it possible to obtain images that clearly delimited the walls of the aorta and its details. The endovascular simulation was then able to be performed, by correctly positioning the endoprosthesis, followed by its deployment.

**Conclusion:**

The 3D printing allowed the construction of aortic diseases realistic prototypes, offering a 3D view from the two-dimensional image of computed tomography (CT) angiography, allowing better surgical planning and surgeon training in the specific case beforehand.

**Table:** 

Abbreviations, acronyms & symbols				
3D	= Three-dimensional		NYHA	= New York Heart Association
An	= Aneurysm		PAU	= Penetrating aortic ulcer
CAD	= Computer aided design		PLA	= Polylactic acid
CT	= Computed tomography		STL	= STereoLithography
DICOM^®^	= Digital Imaging and Communications in Medicine		TL	= True lumen
FDM	= Fused deposition modeling		UV	= Ultraviolet
FL	= False lumen			

## INTRODUCTION

In recent decades, the endovascular treatment of aortic diseases, such as aneurysms, dissections, and penetrating ulcers, became widely used for being a minimally invasive procedure with satisfactory long-term outcomes, acceptable mortality rates, and lower postoperative complications^[1-6]^.

For assisting heart surgeons in the aorta endovascular field, three-dimensional (3D) printing (or rapid prototyping) has become an affordable reality and it is rapidly expanding its applications, both in surgical planning and in education and training of residents and students^[7-14]^.

This technique permits the construction of physical prototypes from conventional medical images, such as computed tomography (CT) scan and magnetic resonance imaging, by converting the anatomical information into computer aided design (CAD) files^[11-13]^.

This process is performed by using a specific software and requires both the anatomical knowledge for selecting the regions of interest and the acquaintance with graphic features to perform the steps.

Therefore, our objective is to present the 3D printing feature on developing prototypes leading to improved aortic endovascular surgical planning, as well as transcatheter aortic valve implantation, and mainly enabling training of the surgical procedure to be performed on patient's specific condition.

## METHODS

From multi-institutional image database, six 3D real scale prototypes were built representing different aortic diseases, taken from real patients, to simulate the correction of the disease with endoprosthesis deployment in the hybrid room.

For the prototype assembly, the image files used were Digital Imaging and Communications in Medicine (DICOM®) CT angiographies obtained in 64-slice CT scanners, using a mechanical injector pump with 4 mL/s contrast infusion speed and isotropic voxels at 0.625 mm.

These DICOM® files were exported to the software Invesalius® (Centro de Tecnologia da Informação Renato Archer, Campinas, Brazil), to target the aortic lumen and its main branches and create a 3D model of the aortic lumen in STereoLithography (STL) format, which is compatible with 3D printing. Then, these files were exported to Fusion 360® (Autodesk Inc.), a CAD manipulation software, to extrude a 2 mm uniform artificial wall, obtaining a hollow file representing the aortic lumen, the luminogram.

Next, these files were sent to 3D printers. In this study, we chose to use two different printing technologies: a fused deposition modeling (FDM) technology printer, the MakerBot Replicator 2®, which uses a temperature extruder nozzle for a plastic filament called polylactic acid (PLA) in red color; and a multijet technology printer, the 3D Systems ProJet 3510®, which employs an ultraviolet (UV) photocurable resin, allowing printing on a semitransparent material.

Finally, the printed 3D models were taken to the hybrid operating room and examined under fluoroscopy, making it possible to obtain images that clearly delimited the walls of the aorta and its details. The endovascular simulation was then able to be performed, by correctly positioning the endoprosthesis followed by its deployment.

### Clinical Cases

**Fig. 1:**
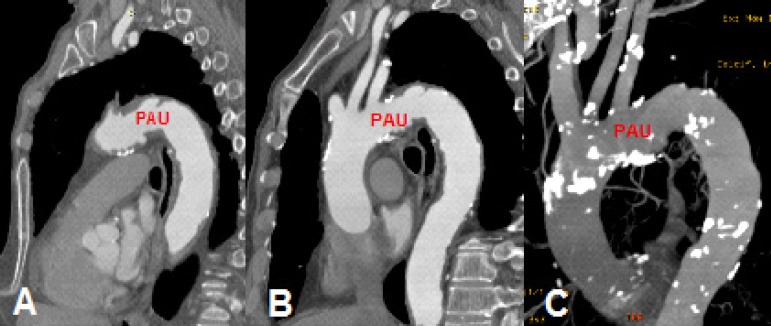
Case 1: computed tomography (CT) angiography of penetrating aortic ulcer (PAU) on the thoracic aorta with a maximal diameter of 57mm. A) Oblique sagittal section on maximal diameter. B) Oblique sagittal section showing its relation to the great vessels. C) Reconstruction of the aneurysm.

Case 1: 65-year-old male, with a penetrating aortic ulcer (PAU) on the descending thoracic aorta. On the CT angiography (Figure 1), it seemed to start right after the origin of the left subclavian artery, extending to the descending thoracic aorta, with a maximum diameter of 57 mm.

Patient was selected for endovascular treatment. The initial planning suggested that the endoprosthesis could be anchored in Zone 3^[15]^ with the free flow zone over the left subclavian artery.

**Fig. 2:**
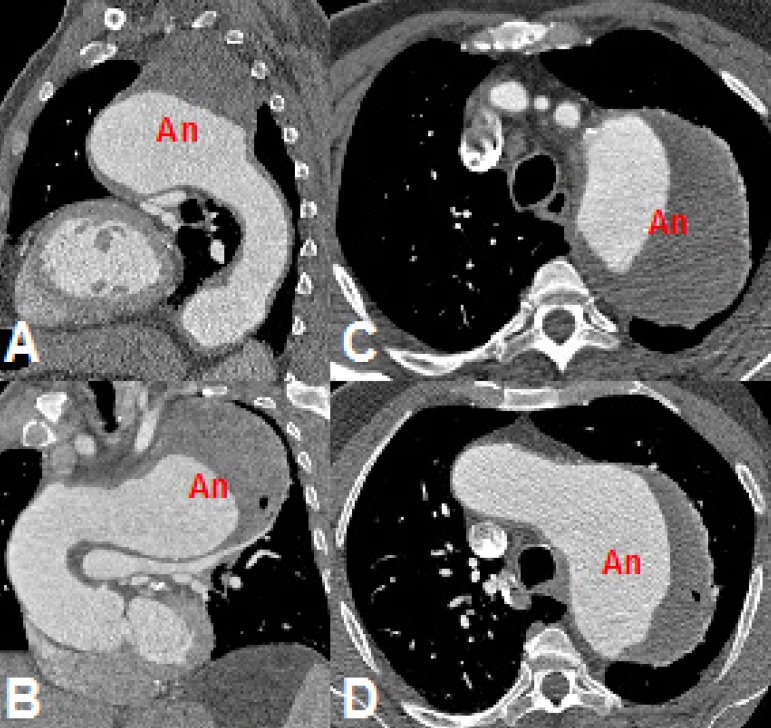
Computed tomography (CT) angiography of the 10 cm thoracic aortic aneurysm (An). A) Sagittal section. B) Oblique sagittal section at maximum diameter. C) Axial section at the level of the great vessels. D) Axial section at the level of the aortic arch.

Case 2: 67-year-old male, with a descending aorta aneurysm and distal involvement of the aortic arch with a maximum diameter of 10.0 x 9.2 cm (Figure 2).

**Fig. 3:**
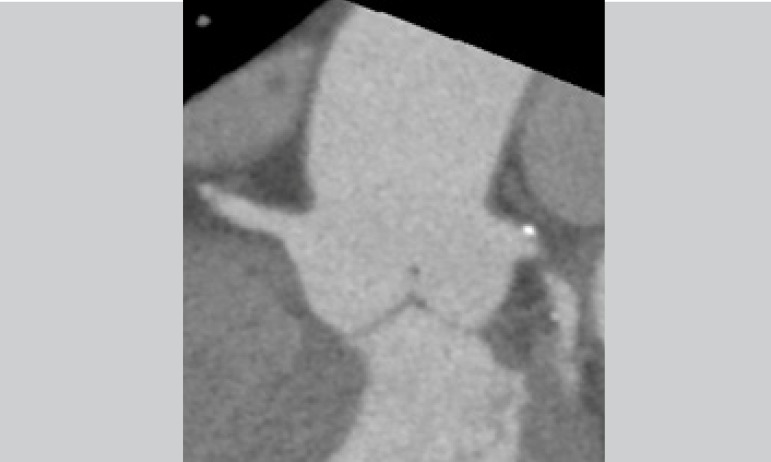
Case 3: computed tomography (CT) angiography identifying the anatomical features of the left ventricle outflow tract.

Case 3: 75-year-old male, being followed-up by the Cardiology team for severe aortic valve stenosis with an ejection fraction of 30% in New York Heart Association (NYHA) functional class III (Figure 3).

**Fig. 4:**
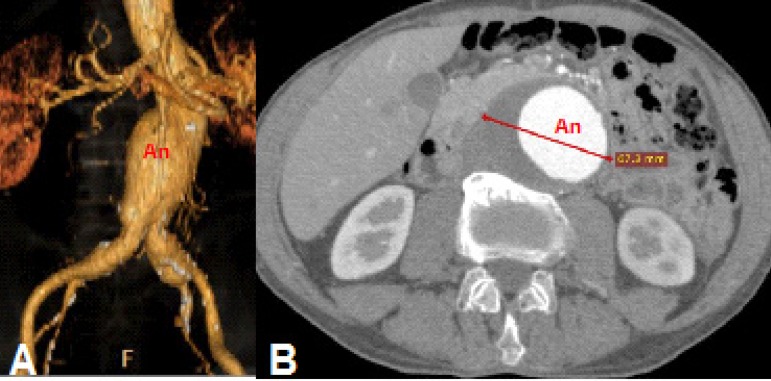
Case 4: computed tomography (CT) angiography of abdominal aortic aneurysm (An). A) Digital reconstruction of the aneurysm. B) Axial section measuring the aneurysm, 67.2 mm in diameter.

Case 4: 68-year-old male, with asymptomatic infrarenal abdominal aortic aneurysm extending to the iliac arteries with maximum diameter of 67 mm (Figure 4).

**Fig. 5:**
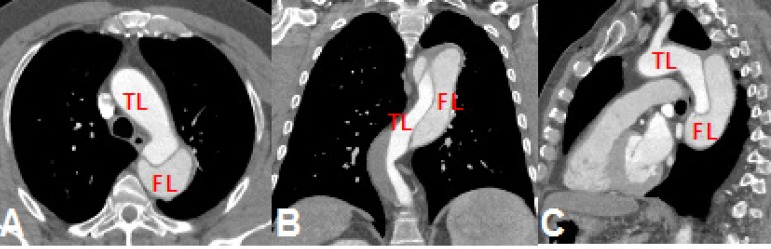
Case 5: computed tomography (CT) angiography of aortic dissection showing true lumen (TL) and false lumen (FL). A) Axial section on the level of the arch. B) Coronal section of the descending thoracic aorta. C) Sagittal section of the aortic arch.

Case 5: 64-year-old male, 3 years follow-up for Stanford type B chronic aortic dissection, with acute abdominal pain, undergoing surgical treatment (Figure 5).

**Fig. 6:**
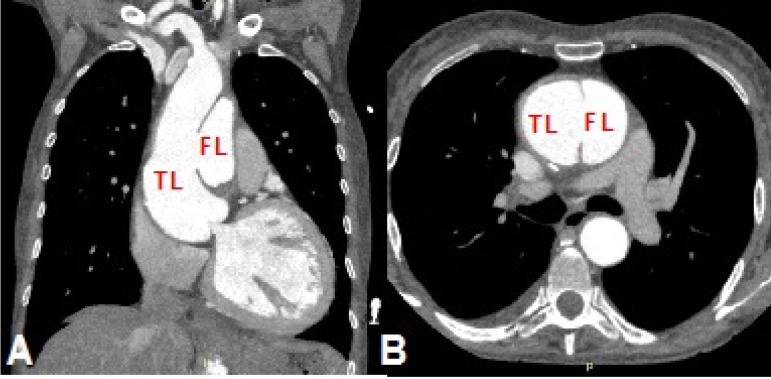
Case 6: computed tomography (CT) angiography of aortic dissection showing true lumen (TL) and false lumen (FL). A) Coronal section showing the dissection in the ascending aorta with its intimal tear. B) Axial section at the level of the intimal tear.

Case 6: 67-year-old female, Stanford type A chronic dissection, with acute dyspnea and chest pain undergoing ascending aorta replacement (Figure 6).

## RESULTS

Using 3D printing, a specific full-scale endoluminal anatomy prototype (luminogram) was obtained in detail in all cases, allowing a proper surgical technique planning, with perfect 3D understandable aorta visualization and revalidation of the endovascular planning.

**Fig. 7:**
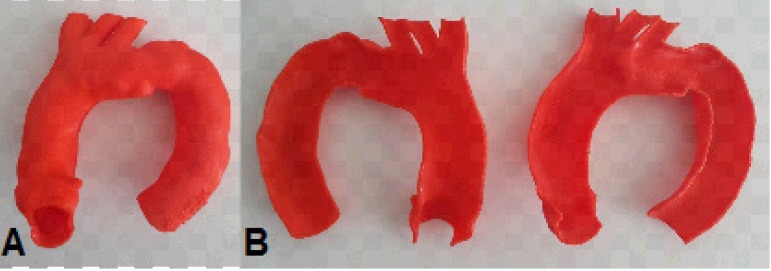
Case 1: prototype printed on red polylactic acid (PLA). A) View from the front, showing the penetrating aortic ulcer in the arch. B) Prototype split, showing the inside view.

**Fig. 8:**
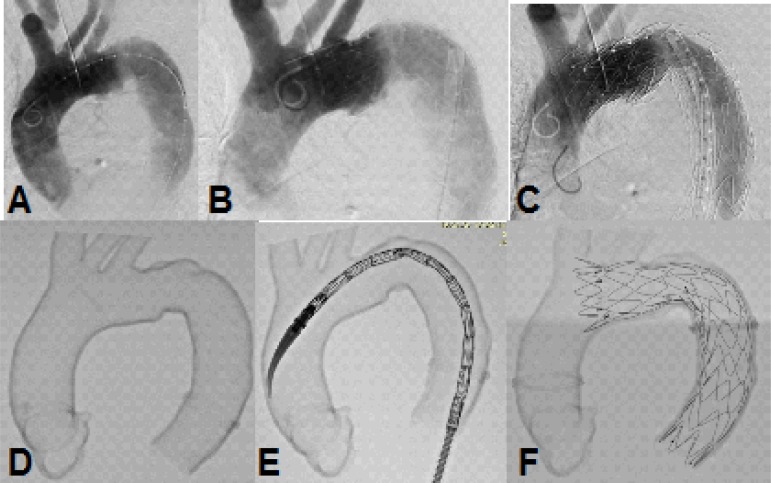
Case 1: A) Fluoroscopy of the patient. B) Failure after deploying first endoprosthesis. C) After second endoprosthesis deployment on Zone 2. D) Fluoroscopy of the prototype. E) Simulation of endoprosthesis deployment on prototype. F) Prototype after endoprosthesis deployment.

In case 1, the prototype showed a difficult proximal landing zone at Zone 2^[15]^ (Figure 7). During the procedure, the endoprosthesis backed off and anchored itself inside the ulcer, requiring an extension anchored in Zone 2^[15]^ with free flow over the left carotid artery for proper treatment, completely covering the penetrating ulcer (Figure 8).

**Fig. 9:**
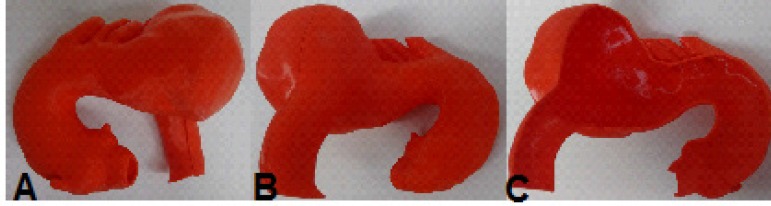
Case 2: prototype printed on red polylactic acid (PLA). A) Frontal view of the prototype. B) Rear view. C) Inside view.

**Fig. 10:**
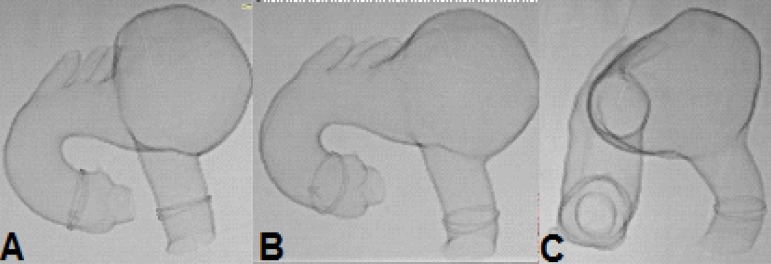
Case 2: fluoroscopy of the prototype. A) On zero degrees. B) 30° left. C) 60° left.

**Fig. 11:**
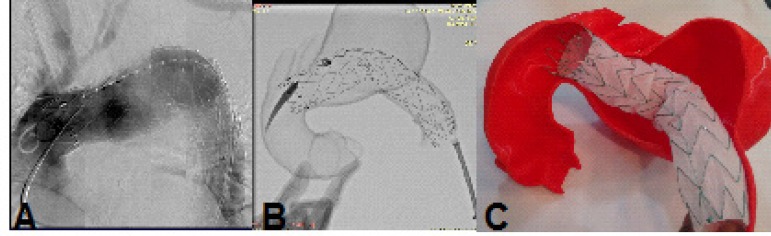
Case 2: comparison between the prototype and the procedure. A) Fluoroscopy of the patient after procedure. B) Fluoroscopy of the prototype after procedure. C) Endoprosthesis inside prototype after deployment.

In case 2, the prototype (Figures 9 and 10; Video 1) showed the need for an additional procedure to create a proximal landing zone in Zone 1^[15]^, either by exclusive endovascular technique, such as the chimney, or by hybrid techniques with extra-anatomical tube or debranching^[16-19]^ (Figure 11; Video 2).

**Video 1:**
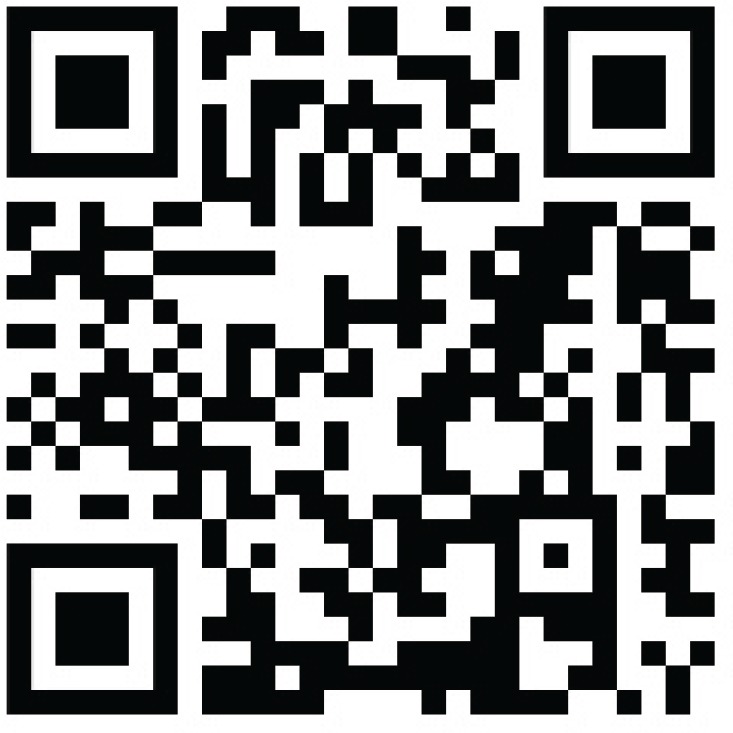
Case 2: fluoroscopy of the prototype.

**Video 2:**
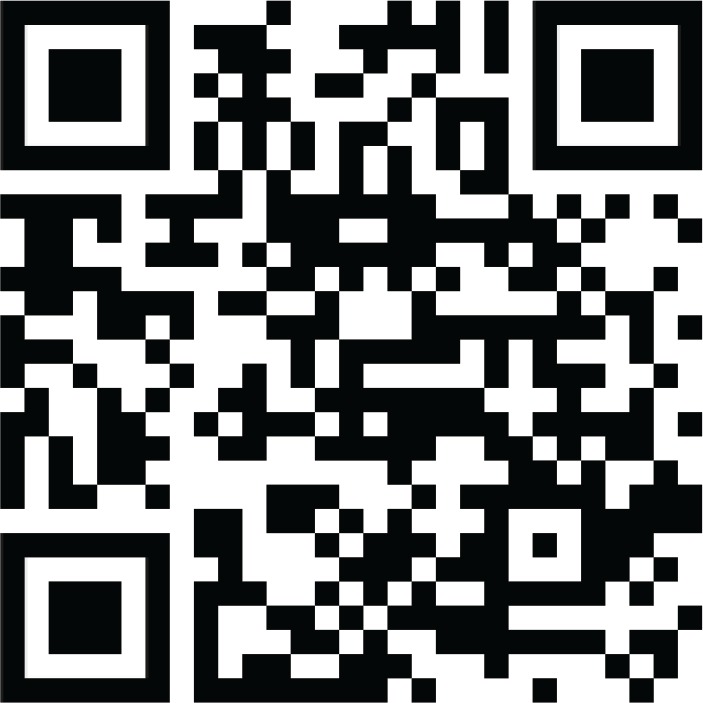
Case 2: simulation of endoprosthesis deployment on prototype.

**Fig. 12:**
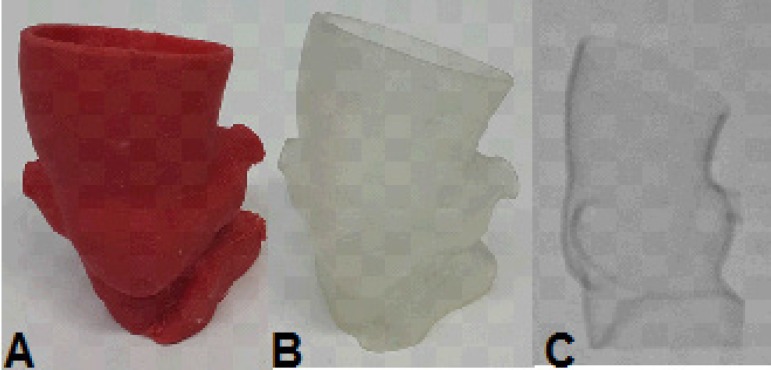
Case 3: prototype. A) Printed on red polylactic acid (PLA). B) Printed on semitransparent resin. C) Fluoroscopy of the prototype.

In case 3, the left ventricular outflow tract was well represented, making it possible to estimate the size of the aortic annulus and the height of the coronary ostia, although it was not possible to reconstruct the aortic cusps. However, this reconstruction limitation did not prevent the prototype's use for preoperative training (Figure 12).

**Fig. 13:**
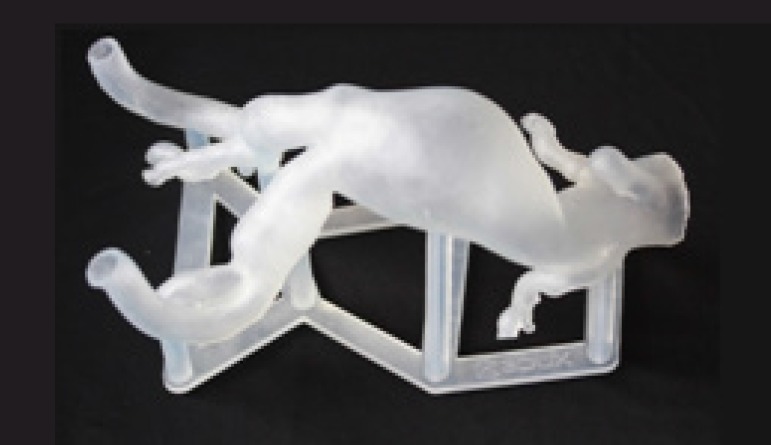
Case 4: prototype printed on semitransparent resin.

In case 4, the abdominal aortic aneurysm 3D prototype (Figure 13) reinforced that the proximal landing zone was a difficult one but it was adequate to release aortic bi-iliac endoprosthesis, due to its angle (Video 3).

**Video 3:**
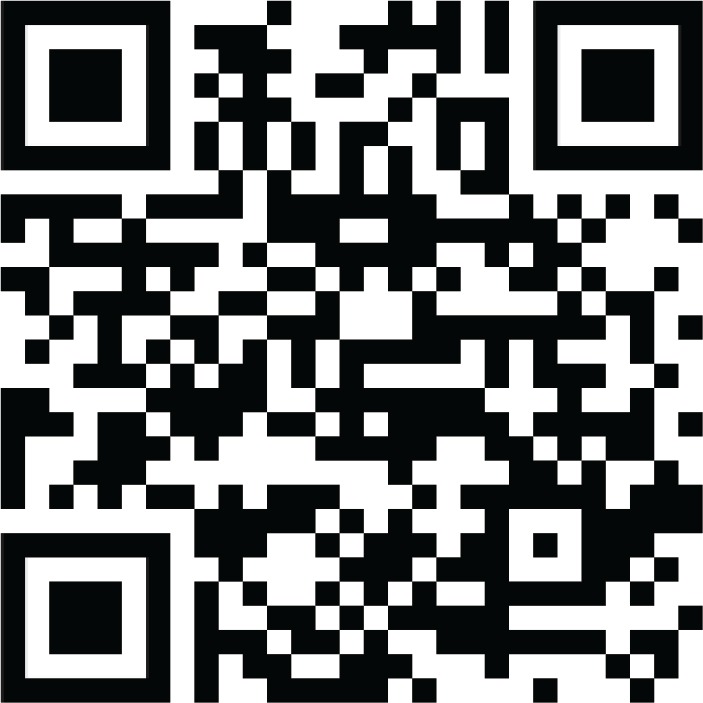
Case 4: fluoroscopy of the prototype.

**Fig. 14:**
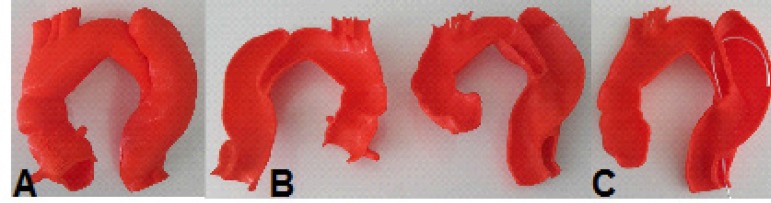
Case 5: Prototype printed on red polylactic acid (PLA). A) Frontal view showing the false lumen. B) Prototype split, inside view showing the dissection. C) Prototype split with wire coming distally from true lumen to false lumen through the intimal tear.

**Fig. 15:**
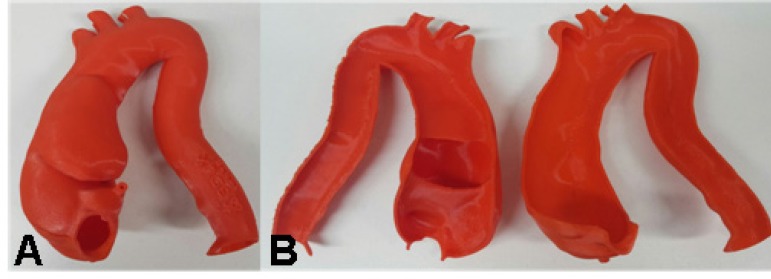
Case 6: Prototype printed on red polylactic acid (PLA). A) View from the front showing the extent of the false lumen. B) Prototype split showing the intimal tear.

**Fig. 16:**
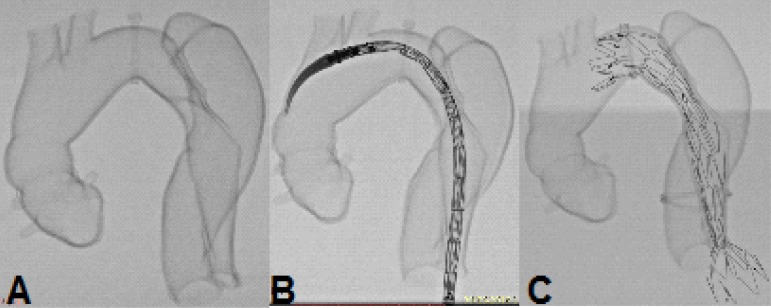
Case 5: simulation of endoprosthesis deployment on prototype. A) Fluoroscopy of the prototype. B) Simulation of endoprosthesis deployment on prototype. C) Prototype after endoprosthesis deployment.

In cases 5 (Figure 14) and 6 (Figure 15), the aortic dissection was well represented, the intimal tear was spotted, and the length of the false lumen was sized. In the type A dissection, the prototype allowed the evaluation of a possible unconventional treatment for this segment of the compromised aorta or an endovascular treatment with customized stent. In type B dissection, the prototype served as a good model for training the identification of true and false lumens (Figure 16).

## DISCUSSION

From our experience, the prototypes used proved to be efficient and reliable for understanding and possibly refining the operative strategy in the assessed aortic diseases.

In recent years, 3D printing technology proved to be a very useful tool in different fields of medicine. Among the various medical applications, orthopedic surgery, otolaryngology, neurosurgery, and plastic and maxillofacial surgeries are the most benefited by the method^[20,21]^. In part, this is due to lower technical complexity in extracting the CAD models from bone structure images, owing to its marked contrast between its limits with adjacent soft tissue structures. Also, because there are many diseases to which the 3D vision and its understanding become extremely essential for a successful surgical treatment.

Recently, a growing number of studies on 3D printing applications have been emerging, enhancing understanding and surgical planning of cardiothoracic diseases, mainly because of technical development and popularization of imaging methods such as CT angiography and cardiac magnetic resonance imaging^[11-13]^.

One of the 3D printing limitations in cardiothoracic diseases is the possibility of errors during the segmentation from CT angiography images to obtain the STL file. This segmentation process is observer-dependent and it requires an extensive anatomical knowledge so that, during these steps, it is guaranteed that all tissues relevant to the case are selected and regions outside the organs of interest and not real structures, such as artifacts, are not represented.

In addition, many scans have satisfactory imaging from a diagnostic point of view, but they are full of shadows and artifacts, preventing the extraction process of the regions of interest in an appropriate manner.

In this study, the main use of 3D printing was to better preoperatively understand the patient's anatomy, especially to accurately comprehend the dimensional scale, angulations, and other elements, as the aneurysmal landing zones and the location of a penetrating ulcer or intimal tear.

A cost-effectiveness assessment of prototyping was not carried out in this study, however, due to its affordable cost, it may prove to be cost effective. First, for allowing to better define the type, length, and oversizing of the endoprosthesis, reducing the chance for further extension. Second, it allows the surgeon to practice the surgery before the actual operation. Thus, at the time of the procedure, the time saving is also considerable due to the high cost of a hybrid room; the reduced use of radiopaque contrast decreases the costs and the insult to renal function, especially in patients with kidney failure; and reducing the use of radiation, the exposure of the patient and staff to its hazardous effect is shortened. And third, the reproduction of knowledge through educational activities or training of other surgeons, residents, and medical students using prototype simulators, which can be used in the hybrid room, can have an impact on reducing the technical learning curve^[22]^.

Particularly, the conditions affecting the aortic arch are more challenging, because of the concern of cerebral protection in case of an open surgery or of maintaining cerebral perfusion by endovascular techniques or a hybrid approach. These conditions can be classified as proposed by Mitchell and Ishimaru^[15-19]^ according to the proximal landing zone of the endoprosthesis. Zones 3 and 4 are routinely treated by endovascular techniques; and Zones 0, 1, and 2 require a more complex approach, either endovascular, hybrid, or conventional. In our service, for landing in Zone 1 or 2, we use the hybrid technique with revascularization of the great vessels (debranching) in order to create a favorable landing zone to deploy the endoprosthesis without compromising the brain and the left upper limb perfusion.

The evaluation of the 3D model may show a more proximal landing zone than predicted by the analysis of CT, as occurred in the cases 1 and 2, requiring a change in the operating strategy, such as an initial hybrid approach (case 2) or the sacrifice of the left subclavian artery (case 1), saving an endoprosthesis extension^[16-19]^.

With these case series, we add more examples of the feasibility of using 3D printing in cardiothoracic surgery, reinforcing its potential benefits in understanding, surgical planning, and also in training and education. In fact, this study is an initial experience to establish a closer contact with prototyping, however, more work is needed in this field to objectively assess the cost-effectiveness, by understanding the case studied, and the possible clinical implications, such as saving endoprosthesis extensions and the possibility of surgical time reduction, along with decreased anesthetic time, contrast, and radiation doses or surgical outcome improvement.

## CONCLUSION

The 3D printing allowed the construction of aortic diseases realistic prototypes, offering a 3D view from the two-dimensional image of CT angiography, allowing better surgical planning and surgeon training in the specific case beforehand.

**Table:** 

Authors' roles & responsibilities	
ENG	The prototypes were designed and constructed; the prototypes were tested; final approval of the version to be published
RRD	Revising it critically for important intellectual content; final approval of the version to be published
BAR	The prototypes were designed and constructed; final approval of the version to be published
JADS	The prototypes were tested; final approval of the version to be published
FJSD	The prototypes were tested; final approval of the version to be published
EKS	Revising it critically for important intellectual content; final approval of the version to be published
WJG	Revising it critically for important intellectual content; final approval of the version to be published
FBJ	Revising it critically for important intellectual content; final approval of the version to be published
